# Glycogen Synthase Kinase 3: A Point of Integration in Alzheimer's Disease and a Therapeutic Target?

**DOI:** 10.1155/2012/276803

**Published:** 2012-06-14

**Authors:** Siddhartha Mondragón-Rodríguez, George Perry, Xiongwei Zhu, Paula I. Moreira, Sylvain Williams

**Affiliations:** ^1^Department of Psychiatry, Douglas Hospital Research Center, McGill University, Montreal, QC, Canada H4H 1R3; ^2^UTSA Neurosciences Institute and Department of Biology, College of Sciences, The University of Texas at San Antonio, San Antonio, TX, USA; ^3^Department of Pathology, Case Western Reserve University, Cleveland, OH, USA; ^4^Center for Neuroscience and Cell Biology, University of Coimbra, Coimbra, Portugal; ^5^Faculty of Medicine, Institute of Physiology, University of Coimbra, Coimbra, Portugal

## Abstract

Glycogen synthase kinase 3 (GSK3) has been implicated in neurological disorders; therefore, it is not surprising that there has been an increased focus towards developing therapies directed to this kinase. Unfortunately, these current therapies have not taken into consideration the physiological role of GSK3 in crucial events like synaptic plasticity. With this in mind we will discuss the relationship of synaptic plasticity with GSK3 and tau protein and their role as potential targets for the development of therapeutic strategies. Finally, we will provide perspectives in developing a cocktail therapy for Alzheimer's treatment.

## 1. Introduction 

Glycogen synthase kinase 3 (GSK3) is an evolutionarily conserved protein that is active in resting cells and is inhibited in response to activation of several distinct pathways such as the Wnt, insulin, and the growth factor pathway [1–7]. GSK3 activity is regulated by different mechanisms, including (a) phosphorylation at an N-terminal serine [7, 8], (b) through phosphorylation of a tyrosine residue [9], (c) through phosphorylation of a C-terminal serine residue [10], and (d) through disruption of the axin-*β*-catenin multiprotein complex [4, 11, 12]. The other requirement of GSK3 is that most of its substrates require prior phosphorylation at residue 4 or 5 amino acids C-terminal to the target residue [13]. 

GSK3 has two isoforms GSK3*α* and GSK3*β*, which are encoded by different genes [14]. In mouse, rat, and human, an alternative isoform (GSK3*β*2) that contains a 13-amino-acid insert near the catalytic domain was reported [15]. In opposition to GSK3*α* and GSK3*β*, GSK3*β*2 is specifically found in the nervous system and has been strongly linked to neurodevelopment [15]. 

In order to participate in all these events, GSK3 has a broad range of substrates: cyclic AMP response element-binding protein (CREB), neurogenin 2, SMAD1, NFkappaB, Myc, heat shock factor-1, cyclin D1, nuclear factor of activated T-cells and CCAAT/enhancer-binding proteins, c-Jun, *β*-catenin, and microtubule-associated proteins like MAP2 and tau [16–18]. GSK3 regulates some of these factors by controlling their protein levels. However, changes in GSK3 activity have been associated with neurodegenerative diseases, such as bipolar disorder, schizophrenia, and Alzheimer's disease (AD) [19]. Indeed, in AD the active form of GSK3*β* was found to be directly related to the hyperphosphorylation of tau present in paired helical filament (PHF)-tau of neurofibrillary tangles (NFTs) [20]. Importantly and due to the fact that most drugs bind and compete with ATP, there appears to be only a single amino acid difference (Glu196 in GSK3*α*, Asp133 in GSK3*β*) making it difficult to identify an inhibitor that can distinguish the two isoforms. 

Overall, it is clear that GSK3 is related to AD development, and, more importantly, current data suggest that both isoforms (GSK3*α* and GSK3*β*) contribute to AD pathogenesis. 

## 2. Tau Pathology and GSK3

Tau is an axonal protein that regulates microtubule stability [21]; however, during AD tau is abnormally phosphorylated and aggregates into NFTs [22, 23]. Tau has at least 45 phosphorylation sites, mostly located in the proline-rich region (P-region) (residues 172–251) and the C-terminal tail region (C-region) (residues 368–441) [24]. Tau phosphorylation at both of these regions affects its capacity to interact with microtubules [25]. In terms of AD pathology, the phosphorylation sites located in the C-terminal region seem to cause (a) abnormal folding and (b) protein cleavage, which together could lead to tau deposition [26–28]. Phosphorylation at some sites (Ser262) selectively impairs binding of tau to microtubules [29], whereas phosphorylation at other sites (Ser202) enhances tau polymerization [30]. Crucially, GSK3*β* has been linked to many of these sites [15, 31]. Therefore, emphasis has been placed particularly on GSK3*β*, rather than GSK3*α*. However, due to the lack of inhibitor's specificity, GSK3*α* has not been ruled out. Indeed, some studies have shown that GSK3*α* through Wnt signalling pathway is also related to tau pathology [32]. Furthermore, by specifically knocking down GSK3*β*, GSK3*α* was found to be related to AD pathology [33]. 

In sum, the current data shows that both isoforms GSK3*α* and GSK3*β* could be involved in tau phosphorylation. 

## 3. GSK3 as the Therapeutic Target for AD

GSK3 is strongly implicated in neurodegeneration [34], and, not surprisingly, it has been postulated as a therapeutic target in the treatment of AD. Indeed, lithium which is a direct inhibitor of both GSK3*β* and GSK3*α* has been used in humans [35, 36]. The direct regulation of GSK3 also modifies cell survival as it is known for facilitating a variety of apoptotic mechanisms [35]. Similarly, in an attempt to reduce tau pathology, the GSK3 inhibitor [Tideglusib/NP-12 (Nypta)] is currently in clinical trial [37]. NP-12 has been designated as an orphan drug by the EU and US authorities and has been granted Fast Track status by the FDA (see http://www.noscira.com). 

The rationale is simple; blocking GSK3 will lead to nonphosphorylated tau and, consequently, less tau deposition according to the current hypothesis. However, the importance of GSK3 for normal physiological cell functioning must be taken into consideration. In this regard, we recently found that phosphorylation of tau protein is critical in order for the protein to function as a negative feedback mechanism to prevent NMDA-receptor overexcitation (unpublished data). This data becomes crucial in this debate since NMDA deregulation plays a vital role in synaptic plasticity. Therefore, by simple blockade of GSK3 we could alter the homeostasis of synaptic plasticity among other important physiological functions. Furthermore, blocking GSK3 also raises the possibility of affecting gene expression and cell survival [17]. So, is GKS3 the desired therapeutic target for AD? Although the answer is far from being simplistic, normal physiological functions for the cell, together with the complexity of the phenomena [38], need to be taken into consideration before selecting AD pharmacological targets. 

## 4. GSK3 as Crucial Node for Synaptic Plasticity 

Synaptic plasticity has been proposed to play a central role in brain capacity to incorporate transient experiences into persistent memory traces. Synaptic transmission can be enhanced (long-term potentiation, LTP) or depressed (long-term depression, LTD) by activity, and these changes can persist from seconds to hours and days [39, 40]. Importantly, the affected intracellular pathways leading to LTP or LTD activation involve primarily GSK3 [41, 42]. Indeed, it has been shown that enhanced GSK3 signalling impairs hippocampal memory formation [43]. Specifically, GSK3 activity blocks synaptic LTP and induces LTD [43]. Furthermore, it was found that GSK3 during LTP involves activation of NMDA receptors and the PI3K-Akt pathway consequently disrupting the ability of synapses to undergo LTD [43]. Clearly, the data claims that GSK3 is a crucial node mediating the LTP to LTD transition. Therefore, the simple idea of blocking GSK3 in order to prevent the progression of AD seems to be overly simplistic. 

## 5. Conclusion and Perspectives

The hypothesis that GSK3 plays a role in the aetiology of brain disorders is further nurtured by the fact that several genetic susceptibility factors for psychiatric disorders have key roles in neurodevelopment. Importantly, many of the genes are involved in GSK3 signaling [44, 45]. Furthermore, GSK3 is directly related to the pathogenesis of AD as tau kinase [31]. Overall, it seems clear that GSK3 has an integral role in the pathogenesis of AD. Therefore, GSK3 remains as therapeutic target. However, the secondary effects caused by GSK3 blockade should also be taken into consideration, especially knowing that synaptic dysfunction in addition to neuronal death can lead to cognitive failure associated with AD. With this in mind, therapies that focus on rescuing events like LTP rather than single blocking strategy could bring needed results. 

**Figure 1:**
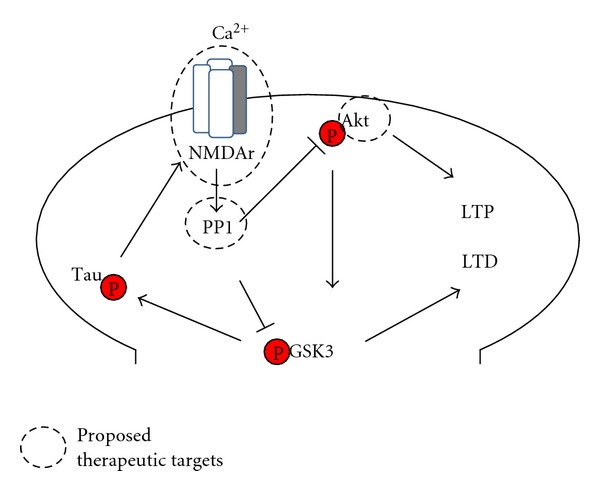
Cocktail drugs could balance the activity of GSK3 during AD. The role of PP1 and Akt in GSK3 activation, in combination with NMDA receptor, makes them important therapeutic targets. Calcium (Ca^2+^) enters via NMDA receptors, and this leads to activation of protein phosphatase 1 (PP1), a key enzyme in synaptically induced LTD. PP1 can dephosphorylate GSK3 that determines whether NMDA receptor activation induces LTD or inhibits LTD. PP1 can dephosphorylate Akt, resulting in GSK3 activation. GSK3, under the control of Akt and PP1, is a critical determinant of the direction of NMDA receptor-dependent plasticity. The active GSK3 isoforms contribute to phosphorylation of tau protein which is essential in order for the protein to function as a negative feedback mechanism to prevent NMDA-receptor overexcitation and synaptic failure.

In conclusion, we suggest that downstream targets of GSK3 are an interesting option. In other words, we proposed the use of *cocktail drugs* that could enhance LTP and reduce induction of LTD. For instance, drugs like memantine (NMDA receptor antagonist) [46], in combination with other drugs like okadaic acid (PP1 activator) [47] and/or pseudosubstrate for Akt [43], could be used in order to balance the activity of GSK3 and therefore tau phosphorylation (Figure 1). Together, this combinatorial approach may result in LTP promotion and synaptic improvement. After all, if the current strategies for AD treatment have shown little benefits, it is tempting to consider new therapeutic approaches that are aimed to improve memory formation.

## Abbreviations

AD: Alzheimer's disease
GSK3: Glycogen synthase kinase 3
NFTs: Neurofibrillary tangles
LTP: Long-term potentiation
LTD: Long-term depression
NMDA: N-methyl-D-aspartate receptor.
